# @Indonesiatanpafeminis.id as a Challenge of Feminist Movement in Virtual Space 

**DOI:** 10.3389/fsoc.2021.668840

**Published:** 2021-09-13

**Authors:** Eni Maryani, Preciosa Alnashava Janitra, Reksa Anggia Ratmita

**Affiliations:** Department of Communication and Information, Universitas Padjadjaran, Bandung, Indonesia

**Keywords:** feminist, anti-feminist, Instagram, virtual space, digital ethnography

## Abstract

The fast-growing social media in Indonesia has opened up opportunities for spreading feminist ideas to a wider and more diverse audience. Various social media accounts especially Instagram that focus on gender advocacy and feminism such as @indonesiafeminis, @lawanpatriarki, and @feminismanis have developed in Indonesia. However, the development of the social media platform also presents groups that oppose feminists. One of the accounts of women’s groups that oppose feminists is @indonesiatanpafeminis.id (@indonesiawithoutfeminist.id). The research objectives are namely to analyze the diversity of issues and reveal the discourse contestation that developed in the @indonesiatanpafeminis.id, and dynamic relationships on the online and offline spaces between groups of feminists and anti-feminists or the other interest. This research employed the digital ethnography method that utilized observation, interview, and literature study as data collection techniques. This study found that the online conversations at @indonesiatanpafeminis.id revealed misconceptions on feminism from a group of women with a religious identity. Furthermore, the conversation also tends to strengthen patriarchal values with religious arguments that are gender-biased. However, the @indonesiatanpafeminis.id serves as a public space for open debates and education on feminist issues. The anti-feminist group behind the @indonesiatanpafeminis.id are women who identify themselves in a certain Muslim circle that has political, cultural, and religious agendas. One of the agendas is to influence the public to reject the Sexual Violence Eradication Bill. This study also noted the Muslim supporters of anti-feminism in Indonesia are less popular compared to progressive religious-based Muslim women organizations such as Aisyiyah (Muhammadiyah), Muslimat NU (Nahdlatul Ulama), and Rahima (Center for Education and Information on Islam and Women’s Rights). The study also evokes discussion on how the feminist and anti-feminist discourses can be utilized to criticize and develop the women’s movement or feminism in a multicultural context.

## Introduction

The presence of the women’s movement on social media attracts attention, especially when the movement is actually against feminism that is trying to fight for women’s interests. This article will discuss social media and the @indonesiatanpafeminis.id (@indonesiawithoutfeminist.id) account that is managed by women but questions the existence of feminists in Indonesia.

Various studies have proven that new media has a tremendous influence on people’s lives in general and in particular for today’s young generation (Balya, Pratiwi, and Prabudi, 2018). Digital media as interactive media allows a reciprocal flow of information that enables users to participate and modify the content of the information at once (Suri, 2019). Therefore, digital media currently has the potential to be used as a means of activism, including gender-based activism or the women’s movement. According to the data from We Are Social, the use of digital media in Indonesia is increasing, reaching 160 million users. Meanwhile, there were 175.4 million internet users in January 2020, an increase of 17% compared to the previous year (Kepp, 2020).

When viewed by gender, a survey conducted by the Indonesian Internet Service Provider Association (APJII) in 2017 noted that female internet users reached 48.57%, while male users amounted to 51.43% (Liputan6.com, 2019). Research conducted by Google Indonesia shows that the number of Indonesian women who can access the internet and digital media in their daily lives is lower than that of men. The reason is because that women have fewer jobs, education, and income (Marboen, 2017; Suwana and Lily, 2017); therefore, women have less space, are underestimated, and are confined to their inability to use technology (Stephani and Kurniawan, 2018). In general, women are very reluctant to participate in digital media, especially social media, especially regarding public issues. They have doubts about their abilities and level of knowledge. Women are reluctant to post on “networked publics” due to their perceptions of emotionally unsafe places (Jackson, 2018).

Consequently, the emergence of social media accounts related to feminist issues from various women groups and organizations is interesting. The mainstream media, such as print, television, and radio, have not accommodated feminist issues (Beck, 1998). This has to do with the perception of media workers who are responsible for producing content. Studies related to news produced by newsrooms found that journalists have negative views on feminists and feminism (North, 2009). Feminists are considered a group of people who have psychological problems, are too emotional, get angry easily, and tend to act extremely (North, 2009). Additionally, one opinion stated that the feminist movement is no longer needed because the work of feminism has been completed (Anderson, 2015). From this point of view, women, regardless of their race, social class, sexual orientation, or their geography, have gained equality in a respectful manner (Anderson, 2015). This view is also developing in Indonesia but it is irrelevant because women’s rights at work are still unprotected and the level of violence against women is still relatively high. Indonesia’s National Commission on Violence Against Women (Komnas Perempuan) noted for 12 years (2006–2019) there was an increase of violence against women around 792%, and many customs in several regions in Indonesia have a high risk for an act of violence against women (Catahu Komnas Perempuan, 2020; Cipta Kerja Law, 2020). Furthermore, the Sexual Violence Eradication Bill has not been passed by the legislative.

The digital feminism movement globally especially came to the foreground after the emergence of the #MeToo movement on Twitter which began in 2017 as a response to acts of sexual violence that occurred in the entertainment industry in the United States. Then within 24 hour, as many as 53,000 people have commented and/or shared the sexual violence they experienced (Kaufman, Dey, Crainiceanu, and Dredze, 2019). The #MeToo movement demonstrates the potential for using Twitter to raise awareness about sexual violence, while providing a space for users to share similar experiences and provide support (Bogen, Bleiweiss, Leach, and Orchowski, 2019).

Meanwhile, in the Indonesian context, the women’s movement has also become increasingly popular, especially since the momentum of the Women’s March Jakarta, which was first held in 2017. Also, some movements respond to sexual violence against children, namely #NyalaUntukYuyun (LightforYuyun) and #YuyunAdalahKita (YuyunisUs) in 2016. These hashtags spread on Twitter as a form of solidarity over the case of the rape and murder of Yuyun, a girl in Bengkulu in April 2016. Owing to a large number of tweets using this hashtag, the hashtag #NyalaUntukYuyun was included in the list of trending topics on Twitter in May 2016 (Supriadi, 2016). Even though it started with Twitter, this hashtag was also used on Instagram for the same act of solidarity. Apart from the solidarity action, the hashtag is also used to advocate for the ratification of legal products, namely the Sexual Violence Eradication Bill.

Apart from Twitter there are some Instagram accounts that support feminism such as @indonesiafeminis, @lawanpatriarki, and @dearcatcallers.id. The emergence of these accounts is important for the continuity of the movement, especially as an effort to provide more space for online discussion. The reason is that the use of hashtags alone is not enough to amplify activism, especially if you want to carry out a revolution (Gerbaudo, 2012). Digital activism through social media accounts such as Instagram can increase the visibility of the women’s movement. Thus, various content uploaded on Instagram can also reach the general public who do not understand the basic concepts of feminism and feminists and their application in everyday life. So, Instagram can act as a means of public education related to gender equality and feminism (Parahita, 2019).

Even though on one hand digital media can be used to expand and develop feminist movements, on the other hand, the platform can also be used by anti-feminist and misogynist groups to resist feminist ideology. Anti-feminists can be understood as a response to gender political values that do not only involve women (Ging and Siapera, 2019). Anti-feminist groups seek to reaffirm control over social and biological reproduction by reducing women to the extent of their reproductive organs and their role as mothers. This movement is in opposition to activist groups fighting for women’s rights (Marshall, 2013). Anti-feminism can be seen as a response to social change and a new pattern of politics in the postindustrial era. Walby (in Cupać and Ebetürk, 2020) sees anti-feminist as a more resistant movement to reaffirm, maintain, and increase the subordination of women by patriarchal forces. The anti-feminist group enters the international area because that is where the threat of patriarchal values, status, identity, and power was coming from now. The transnational anti-feminist mobilization has been particularly well coordinated in Europe.

In Southeast Asia, the anti-feminist movement has been politically strategic because they often positioned human rights are unethical to Asian values (Bong, 2016). The lived realities between men and women in Southeast Asia, in particular their sexual and reproductive health rights, remain greatly influenced by cultures and religions. In Indonesia, Malaysia, and Brunei Darussalam, these realities are influenced by Islamic cultures. In terms of Muslim women’s/feminist activism in Southeast Asia to politicize spirituality is what Rinaldo (2013) terms as “pious critical agency” or the interpretation of texts and “pious activating agency” which deploys religious texts to make claims for political and social change.

One of the frequently mentioned complicating factors in feminism discussion in Indonesia is the assumption that feminist ideas, thoughts, and movements do not have social and cultural roots in Indonesia’s society, or in other words, those ideas and thoughts originated from the West or are seen as having Western connotation (Arifia and Subono, 2017). The term feminism is considered by Islamic woman activists in Malaysia and scholars in China to be “something” from outside “themselves.” They rather changed the term to “womanist” and “feminology.” It is not easy to accept new ideas, especially when the term “feminism” and the concept of gender originally come from the West (Nurmila, 2011). Indonesia, as the home for the largest Muslim population in the world, may have a different view about how to see and accept feminism as one of the concepts of gender and it is led to the rise of anti-feminist groups in Indonesia, especially in social media.

The most prominent Instagram account that promotes anti-feminist content is @indonesiatanpafeminis.id and @lawanfeminisme. Their contents are mostly based on the idea that feminism and feminists are ideas and groups that threaten women’s existence in society instead of fighting for women’s rights. They not only use idealized concepts that come from patriarchy but also relate them to their religious understanding, in this case, Islam as the religion of the majority of Indonesians.

In general, Indonesian Muslims can be divided into two categories, those who accept and adopt Muslim feminism, and those who reject and counter Muslim feminist publications (Nurmila, 2011). This condition has also contributed to the development of Muslim feminists in Indonesia which began in the 1990s. They argue against the use of religion to justify the subordination of women by reinterpreting the Qur’an from a gender equality perspective. One of Indonesia’s Muslim feminist figures is Lies Marcos. Lies, together with other Muslim feminists from NU (Nahdlatul Ulama—the largest Muslim organization in Indonesia) such as Kyai Hussein Muhammad (the Muslim opinion leader), founded Rahima (Center for Education and Information on Islam and Women’s Rights) a nongovernmental organization that supports women’s rights in Islam (Nurmila, 2011). In addition to facing the East and the West, conflicts that are more global, the feminist movement, including Muslim feminists in Indonesia, also experience challenges from politics at the national level. Some Muslims in Indonesia who accept the idea of feminism were the products of western ideology and thought; therefore, those Muslim groups are rejecting or reluctant to support feminism in Islam (Nurmila, 2011).

The authoritarian regime of the New Order with the leadership of Soeharto from its inception took many victims related to political feuds and also used violence as a central strategy to maintain political control and legitimacy of violence on behalf of the state (Ghoshal, 1979; Douglas Wilson, 2006). Apart from suppressing groups with different views, the New Order regime also had very high suspicion of Muslim groups so that the pressure on them was also quite strong and silenced them. After the 1998 reformation, Indonesia experienced a lot of turmoil and presented various groups that were previously silenced who then began to speak out, including the so-called political Islamic group (Abdullah, 2014). As a country that has a majority Muslim population, even though the founders of the Indonesian nation have determined that Indonesia is a republic and not an Islamic state, but religious-based political efforts cannot be avoided which enriched politics in Indonesia (Douglas Wilson, 2006). In addition, with the legacy of ethnic-religious violence, economic depression, and bureaucratic corruption left after Soeharto’s fall from power in 1998, Islam is increasingly attracting many Indonesians as a source of moral stability (Stein, 2007).

Thus, feminist challenges from Islamic groups that were initially silenced are also now developing. The anti-feminist attitudes, which have their roots in male domination both culturally and religiously, are adopted by Muslim women’s groups. These women’s groups not only perpetuate patriarchal values but also ignore the struggles of Muslim women’s groups that support the feminist movement, either known as Muslim feminist groups or Muslim women’s groups based on Islamic organizations in Indonesia. There are two groups of Muslim women based on the largest religious organization in Indonesia, namely Muslimat NU that is part of Nahdlatul Ulama which is known as a traditional Islamic group, and another group named Aisyiyah from Muhammadiyah that represents a moderate Islamic group.

While other studies focused on the digital feminism movement (Nurmila, 2011; Parahita, 2019) and the offline feminism and anti-feminism movement in Indonesia (Djoeffan, 2001; Olviana, 2017), this study observes the opposing group that is @indonesiatanpafeminis.id and specifically investigates its online–offline discourses. The study of @indonesiatanpafeminis.id is the first attempting to understand the discourse of counter, or anti-feminism, on social media voiced by women in Indonesia with the majority of its citizens identified as Muslims. Therefore, this study can raise the potential of social media being a platform in building marginalized discourse in media about feminism. Also, this research serves as a challenge for researchers of gender media studies to acknowledge the women’s presence and listen to their arguments and disagreements.

This article examines the discourse contestation in the @indonesiatanpafeminis.id (@indonesiawithoutfeminist.id) contents and conversations analyze the diversity of issues of the contents and conversations, as well as the dynamic relationships on the online and offline spaces between the feminist and anti-feminist groups. This study also analyzes the development of anti-feminist women’s groups in the public space and the relationship between their online content and offline political discourses in Indonesia.

## Methods

This study employed a qualitative approach with the digital ethnography method to reveal the various elements that represent real-life culture through the unification of characters from the digital media (Underberg and Zorn, 2013). Digital ethnography invites researchers to consider how we live and conduct research in a digital environment and the consequences of the presence of digital media in shaping our techniques and processes in conducting ethnography (Pink, 2017). Through ethnography, researchers try to understand patterns of relationships, behaviors, and sequences in the digital environment (Kaur-Gill and Dutta, 2017). To achieve this goal, this study will focus on self-identity, social relations, and the structure of the digital environment related to the reality being studied (Pink, 2017). This research focuses on activities in the digital space of @indonesiatanpafeminis.id (@indonesiawithoutfeminist.id) and various texts that are produced in the digital space from their activities or other digital activities linked to it.

The Instagram account @indonesiatanpafeminis.id is an Instagram account that opposes the existence of feminism in Indonesia. This account is important to research because it openly and firmly expresses its opposition to feminists compared to other similar accounts such as @ailaindonesia (family love alliance) whose contents also attack feminists. This account also gave birth to other accounts with names that identify big cities in Indonesia where they operate, such as @jakartatanpafeminis2, @padangtanpafeminis_, and @bandungtanpafeminis. The @indonesiatanpafeminis account has 5,082 followers and has been uploaded 119 post (Azmi and Bachri, 2019) before the account disappeared and was replaced by the @indonesiatanpafeminis.id account. The @indonesiatanpafeminis.id (@indonesiawithoutfeminist.id) sent their first post on October 31, 2019. As of December 2020, the account had 1,075 followers and sent 33 posts. The account also follows 18 other accounts, nine of which are affiliated with the @indonesiatanpafeminis.id.

Data in this study were collected through observing activities in the @indonesiatanpafeminis.id, and then examining the virtual interactions of these activities as a virtual space community. Researchers also conducted interviews with practitioners, feminist movement figures, feminist studies experts, political observers, and digital media observers to understand the context and the differences in values, beliefs, and behaviors that appear online. We interviewed a feminist studies expert from Nation Islamic University, a feminist movement figure from Indonesia National Commission on Violence Against Woman (Komnas Perempuan), and a political observer. We explored their points of view toward the anti-feminist movement and its connection with a political interest in Indonesia, through interviews. Furthermore, we interviewed a digital media observer to understand the potential of digital media as a public space. We interviewed three netizens who support and oppose feminism in the @indonesiatanpafeminis.id account to know their opinion about @indonesiatanpafeminis.id.

## Result

Recently, new directions in feminism have emerged due to new technologies, particularly social media that have been recognized as the “fourth wave” feminism (Munro, 2013). Research conducted by Swank and Fahs (2017) identifies factors that can contribute to women’s involvement in politics, or more specifically in changing women from feminist sympathizers to feminist activists (Parahita, 2019).

@indonesiatanpafeminis.id (@indonesiawithoutfeminist.id), as an anti-feminist movement in the digital space, produces contestation between pro-feminist and pro-antifeminist groups in the comment section of the @indonesiatanpafeminis.id Instagram account. In addition, this article will also reveal the relationship between the anti-feminist activities in the offline and online spaces through the presence of @indonesiatanpafeminis.id.

### The Presence of @indonesiatanpafeminis.id (@indonesiawithoutfeminist) in the Virtual Space

Social media users have more open space in expressing their opinions that can be accessed by many people from numerous social groups with various views and targets related to their activities on social media. Based on observations toward @indonesiatanpafeminis.id (@indonesiawithoutfeminist.id) Instagram account, the images of the anti-feminist group were obtained. The first image relates to the identity of women’s groups who play a role in various @indonesiatanpafeminis.id activities. Second, we found diverse issues online on feminist and anti-feminist which developed offline in the Indonesian society.

The identity of the @indonesiatanpafeminis.id account raises questions. The @indonesiatanpafeminis.id account has been a frequent topic since the issue on the Sexual Violence Eradication Bill (Penghapusan Kekerasan Seksual—PKS—Bill) supporting LGBTQ coming out. They opposed the ratification of the PKS Bill and introduced the hashtag #UninstallFeminism in March 2019. This campaign movement was quite massive on Instagram, their goal was to reject feminism developing in Indonesia while simultaneously campaigning for the hashtags (Azmi and Bachri, 2019). According to Azmi and Bachri (2019), the @indonesiatanpafeminis account has 5,082 followers and uploaded 119 posts before the account disappeared and was replaced by the @indonesiatanpafeminis.id account. Various posts uploaded by the @indonesiatanpafeminis account rejected the existence of feminism in Indonesia which is considered inconsistent with religious norms and rules, especially in Islam. They also introduced the hashtags #IndonesiaTanpaFeminis, #antifeminist, and #antifeminism.

Although the @indonesiatanpafeminis.id account does not directly state that their rejection of feminism comes from the teachings of the Islamic religion, some of their posts often link issues on feminism with Islamic teachings. The administrators appear to be a group of Muslim women, this was derived by the garment they wear (the hijab) and by their texts quoting verses from the Quran as well as how they explain issues in the content they uploaded. As a Muslim group, they seem to cover up their identity or what kind of group or organization they are from.

The most viewed upload is the first post to feature animation from one of the administrators. The animation shows a hijab woman speaking in her real voice. She teased feminism as those who could not restrain their lust. It is also supported by the caption, which said, “After this, the feminist who has no clue will start ranting in this comment column. Hahaha.” This upload was viewed more than 12,789 times and became the most popular upload from the @indonesiatanpafeminis.id account.

On the contrary, some accounts from Islamic groups that support the feminist or women’s movement have made their identity open to the public. One of them is the @aisyiyahpusat account that consists of a group of Muslim women who are part of Muhammadiyah. Muhammadiyah is the second-largest Islamic organization in Indonesia after Nadhatul Ulama (NU). The @aisyiyahpusat account is the official account of Aisyiyah which is managed by the Central Leadership Media Team of Aisyiyah. This account is one of several accounts that support women’s equality and has developed a friendlier Islamic approach to women’s equality. They provide various information about families, children, as well as women’s issues. As of December 2020, @aisyiyahpusat has about 27.800 followers and has uploaded 1,538 posts.

In addition to discovering how the identity of the @indonesiatanpafeminis.id account administrator is disguised, this research also produces data on various issues that arise in the contents that were uploaded by the @indonesiatanpafeminis.id before February 2021. Issues in the contents can be interpreted as misunderstandings of various concepts in feminism and feminists, their interpretation of Islam that is gender-biased, and references to patriarchal cultural values in society that are gender-biased or subordinated women.

The actors that appear in the @indonesiatanpafeminis.id account consist of three main categories, namely female heroes, feminist figures, and Muslim women. Emerging female heroes such as RA Kartini and Rahma El Yunusyyiyah are portrayed as women who fight for women’s rights, but they do not agree with female figures being categorized as feminists. Other actors are feminists whose existence they accuse and even harass. They also said that some of the existing feminists were hard-line feminists, although they did not explain what that means. They also seem to think that Muslim women or as they are called Muslimah (the term for female Muslim) must always agree with them. Finally, the last actor who appears is a man, a social media influencer, showing up in a video that accuses, questions, and harasses feminists and even the very position of women. The male actor is shown with several women—not in an animated form—holding a paper with the words “Indonesia is peaceful without feminists.”

**FIGURE 1:**
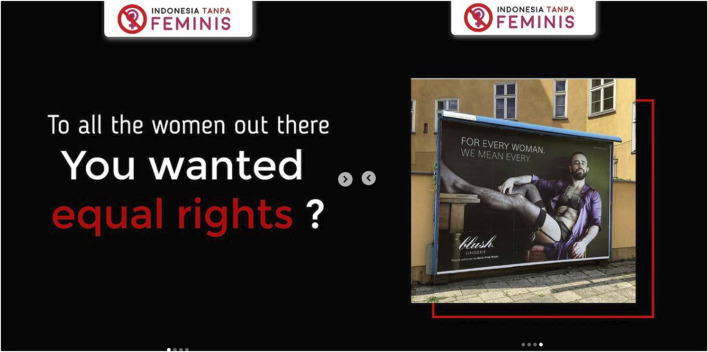
The mockery toward the idea of equality (Posted by @indonesiatanpafeminis.id on July 06, 2020).

The issues that both feminists and @indonesiatanpafeminis.id raised in their content include issues that fight for women’s rights and equality in various fields, especially education. But their posts are contradictory to one another as if what the feminist movement is doing is unneeded or goes against eastern religions and cultures (Indonesian society). The issue of religion was raised by the @indonesiatanpafeminis.id account that linked various feminist views with Islamic views based on their interpretation of women in Islam. These contents say that feminism was born in the West and is not in accordance with the Islamic perspective on women. According to them, in Islam, women have always been glorified so feminism is not needed. In addition, they also interpreted that women have certain limitations, rights, and obligations that they must obey which differentiate them from men. This contradicts the feminist idea that women are equal to men. This view is expressed in posts questioning equality for women and ridiculed the idea by displaying photos of men wearing women’s underwear (Figure 1).

**FIGURE 2:**
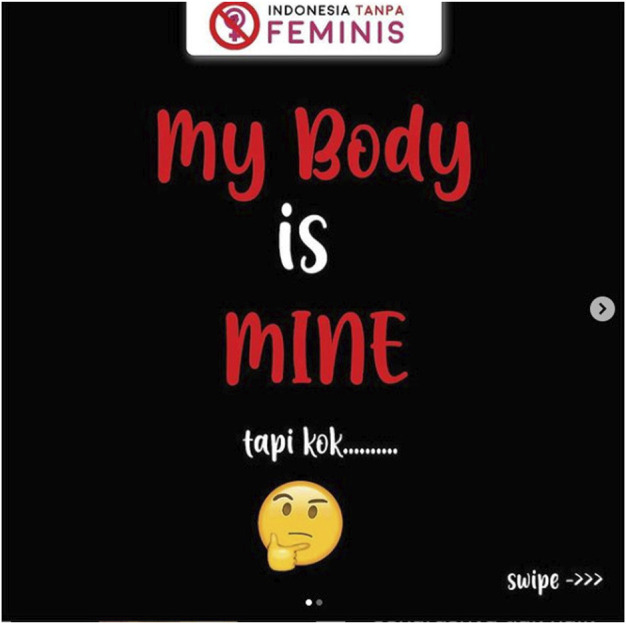
The ownership of women’s bodies issue and inconsistency of feminism (Posted by @indonesiatanpafeminis.id on June 17, 2020).

Add Figure 1; There is a text that said “my body is mine, but …” (Figure 2) and it also contains a statement about body shaming toward women that use hijab or veil. They considered this action as feminist inconsistency which is seen from the statement “my body is mine.” Based on this statement, feminists should not be questioning or mocking the hijab because it is Muslim women’s right.
Add Figure 2; The content regarding violence against women uploaded by @indonesiatanpafeminis.id is contradictory to the context of violence against women and is based on presumption. They stated that women who are the victims of sexual violence could be women who are hurt by their partners so they play victim even though they already give their consent. This opinion could be seen as an effort made by @indonesiatanpafeminis.id to defend sexual violence toward women perpetrators by attacking the victims. They think that when women become a victim of sexual violence, it happens because of their own mistakes. They also ignore the possibility of coercion that happens in sexual violence.

**FIGURE 3:**
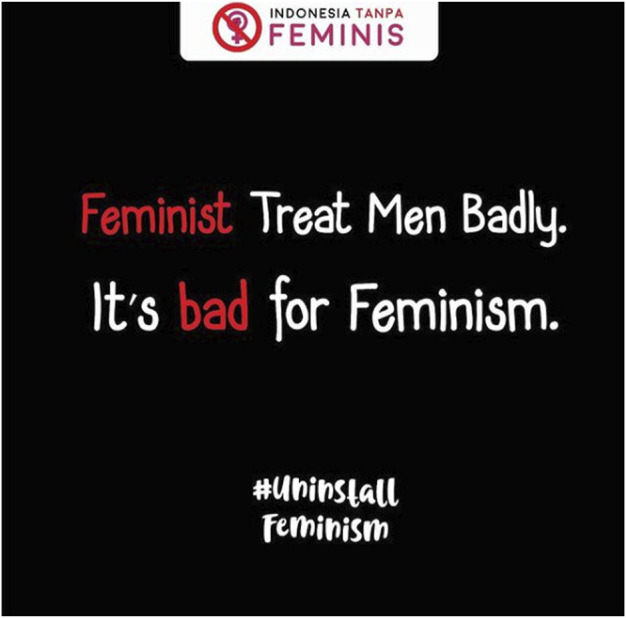
The issue of feminist hatred toward men (Posted by @indonesiatanpafeminis.id on July 29, 2020).

The meanings of the contents promoted by @indonesiatanpafeminis.id about feminists are contradictory and are based on a presumption about feminists in Indonesia. They interpreted feminism as a form of women’s refusal to have a family, refusal to take care of a household and children, as well as women’s hatred and bad treatments toward men (in Figure 3).

Add Figure 3: The @indonesiatanpafeminis.id also stated that there were inconsistencies in the feminist community where women still chose to marry men, this is considered inconsistent with women’s hatred toward men and their families. Another meaning given to feminism that often appears in these accounts is that feminism hates religion because it limits women’s freedom and feminism hates women because feminism idolizes the role of men so much that it forces women to do the same things regardless of women’s physical limitations.

Then regarding the role of a wife, they are contrasting the positions of wives with prostitutes. They asked the public to choose a side between team wife or with team prostitute, gamification of identity in social media. In connection with the meaning of feminism that hates family, the @indonesiatanpafeminis.id stated that feminism hates the family because feminism does not feel the function of the family itself. After all, feminists lack affection and reject the role of men in the family so that they hate the family and therefore oppose patriarchy.

The last theme raised by the @indonesiatanpafeminis.id account is the theme of supporting feminism for the LGBTQ community. Their statement related to feminism and LGBTQ conveyed was that feminists who support LGBTQ should be LGBTQ. Therefore, if a feminist woman who supports LGBTQ is not a lesbian, it means she is a hypocrite because her sexual orientation betrays what she says. Contents posted in @indonesiatanpafeminis.id challenge the statement written from Jurnal Perempuan (The Indonesian Feminist Journal), issue 58, page 14, which contains the words “lesbian in western feminism ideology is the highest achievement of a feminist because women no longer depend on men for sexual satisfaction.” The post received various comments and among them doubted the truth of the quote.

**FIGURE 4:**
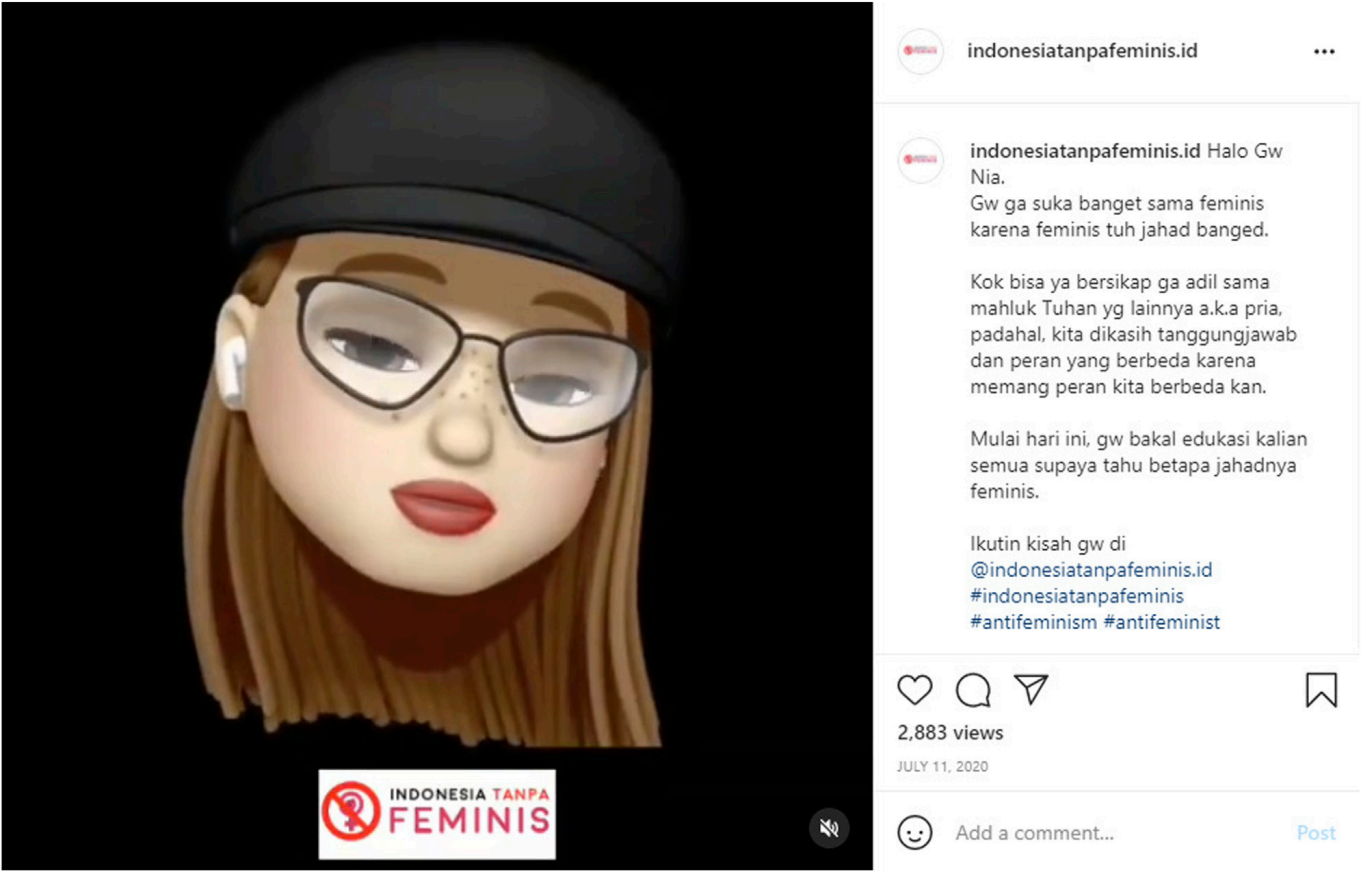
The admin’s profile who does not use hijab (Posted by @indonesiatanpafeminis.id on July 11, 2020).

Related to the hijab, or also known as jilbab, issues in Indonesia, @indonesiatanpafeminis.id uploaded a post that stated “people’s hijab is being taken care of” (posted by @indonesiatanpafeminis.id on February 17, 2021). It means that they accuse people who are making a fuss about the use of hijab, not people who do not use hijab. This statement is also in line with one of their posts that show Nia, one of the admins, who is not wearing hijab (Figure 4). This is interesting because the hijab is often used by them as their own identity.

Add Figure 4: Besides the animation video, this post has also contained a caption: Hi I am Nia. I hate feminist because feminist is evil. How can they be unfair toward other God’s childlike men even though we have responsible and different roles? From now on, I will educate you guys to know how bad is feminists. Follow my story in @indonesiatanpafeminis.id #indonesiawithoutfeminist, #antifeminism, #antifeminist.

### Discourse Contestation on @indonesiatanpafeminis.id

The increasing popularity of social media affects increasing social activism in social media (Shirky, 2011). In line with the development of the use of social media by various groups, social activism in Indonesia also involves various groups. Our observations toward @indonesiatanpafeminis.id (@indonesiawithoutfeminist.id) found the diversity of groups involved in feminist issues from an anti-feminist perspective. In addition, in every post on the @indonesiatanpafeminis.id account, we can also find various hashtags of related issues and mentions to other groups, either with the same or with different views such as @indonesiaperlufeminis.

“Fight feminist thinking! And support @indonesiatanpafeminis. … In frame: women who want not to be exposed to the poison of feminism”. This sentence is written on @indonesiatanpafeminis’s Instagram account in 2019, accompanied by a photo showing dozens of women wearing hijabs in a room with their fists clenched. Based on the analysis of the context in which it appeared, it is known that the @indonesiatanpafeminis account emerged after heated issues regarding the rejection of the Sexual Violence Eradication Bill (PKS Bill). However, this account did not last long, it disappeared in line with the postponement of the discussion of PKS Bill in the parliament. We consistently found that the existence of this account involved the political feud in the parliament related to the endorsement and rejection of the PKS Bill.

#### Equality: Men vs Women

The issue of gender equality is an issue that has been touched on in the debate triggered by a post from @indonesiatanpafeminis.id. Seven posts raised the issue of gender equality. The six of which received 2,547 comments and 1,091 likes and one post in the form of a video received 2,576 views. Related to gender equality, this resulted in various debates that raised issues about the role of men as head of the family, pregnancy, the roles of men and women, and education. One of the equality issues raised by @indonesiatanpafeminis.id is their disagreement about women’s equality which is interpreted as rivaling men, against nature, or disproportionate. This is refuted by a statement containing the view that the issue of gender equality is an effort to fight for the rights that a person deserves, both men and women, not a matter of who is more powerful or against human nature. The issue of equality also presents a statement that Islam guarantees equality for women. This is precisely what is then stated by the other user that there are still so many women who get injustice, even the number on violence against women cases is still relatively high. The comments are very numerous and dominated by statements criticizing the statement about equality in the @indonesiatanpafeminis.id posts.

#### Feminist Versus Family and Religion

The debate also occurred on the issues about family and feminists with five posts, 847 likes, and 1,357 comments. This issue emerged from an article written by an Indonesian female figure, Rahmah El Yunusiyyah, who said that women’s emancipation was no longer needed in Islam because Islam guaranteed women’s rights, especially in the family. One of the posts about family hatred is a video explaining the ideal family that functions as a comfortable and safe place. The post received 10,902 views and 700 comments. Most of the comments came from users who argued that not all families are comfortable and safe places because, in fact, many families are even the most dangerous places, especially for women. One of the comments from pro-feminist circles regarding this statement is “The meaning of feminism: feminism is a movement and ideology that fights for equality for women in politics, economics, cultures, privacies, and publics. Feminism is not an ideology where women hate men.” Regarding the family issue, one of the issues raised was the polygamy case, stating that Kartini (1879–1904) as an Indonesian National Woman figure, who promoted gender equality, also underwent polygamy. This post then received a response that Kartini was a victim of culture which she opposed and hoped that Indonesian women would not suffer the same fate as her. Many comments questioned whether the admin of @indonesiatanpafeminis.id had done in-depth research on Kartini. The post about Kartini received 179 likes and 235 comments.

#### Violence Against Women: Victims Versus Perpetrators

There is a view that all feminists are considered to support free sex which is interpreted as sexual relations based on lust even though this issue is conveyed indirectly, namely in the form of satire. This is shown, for example, through a woman in an animated video stating that what distinguishes humans from animals is their control over lust. The parables taken and the innuendos made are related to hate speech in Indonesia, which calls people who live together without being married *as koempoel gebouw* (cohabitation)*.* Another satirical post appears in the form of a question about the consent given when having sexual intercourse because women are considered too often to play victims when they are hurt, even though they initially gave their consent to have sex freely. This post, which received 190 likes and 250 comments, invited many pros and cons. In another post, there is an insinuation that feminists cannot contain their lust so they engage in casual sex. The post received 12,814 views and 399 comments**.**

The various debates that occurred in @indonesiatanpafeminis.id regarding the issues posted by admins between those who support and those who reject the admin’s opinion or interpretation could be seen in Table 1.

**TABLE 1:** Discourse on interactionist between pro versus anti-feminist in @indonesiatanpafeminisa.

Main issue and content	Pro comment	Contra comment
**Equality: Men vs Women**		
“Equivalent? Equal in what way? When I become the leader of the household, do you want it too? When you get pregnant, should I get pregnant? When you give birth, do I have to give birth? (Posted on November 31, 2019)	“You want to be equal? Do you brave enough to do an MMA fight with men? It’s already good enough that women are being glorified, still, you want to be equal. Weird.”	“The goal of it is gender equality not change the roles of men and women. Equality doesn’t mean that all men’s roles should be women’s and vice versa. What being fought for here is all people deserve to get their rights and no gender is above others.”
**Hijab-sexual Harassment**		
Sexual harassment? Oh boy, what do you think? You all can’t even value your bodies. Don’t you see Islam respects women by having their bodies covered with hijab? (Posted on November 31, 2019)	“Dear admin of @indonesiatanpafeminis.i d, how could they demand respect with their scampy clothes, while those who wear hijabs and niqabs are judged as close-minded”	So you think that hiding our bodies will not make United States target of rape? Will not be catcalled? There are many stories about women in niqab that still became victims of cat- calls, many hijabi women are victims of rape. It’s not about what we wear but it’s about men’s desire
**Polygamy**		
“In a book published by Abendanon, it explained that Kartini was against polygamy. While Kartini was the 4th wife and clearly, she was in polygamy. If she was a feminist why did she agree to be a polygamist?” (Published April 22, 2020)	“Hey admin, they see history as if they are hallucinating @indonesiatanpafeminis.i d … reject them. They run in circles with their ‘oppression of women’”	“That is the reason why she (Kartini) was against it, she was a victim. The core of this topic is that polygamy practice makes women and children feel more miserable. In the Al- Qur’an the context of it is not from nothing to something, but from not controlled to controlled. Along with the times, if people think that polygamy practice is not a good thing, it is okay not to do it”.
**Rejection of Feminism**		
“When a Muslimah is accused of being a feminist. Don’t stay quiet sis. Indonesia is peaceful without feminists.” (Posted on December 10, 2019)	I'M SO GLAD I FOUND THIS PAGE!! In the United States feminism is like poison, not right. We must educate women BUT NOT BY FEMINISM. How can women and men be equal, doesn’t make any sense	“You have the right for expressing your own opinion, we are already independent, but do not manipulate historical facts and make your opinion as to the most correct fact. Even though you are bullied by others, your determination is too strong to think logically. If you think Indonesia does not need feminism, please give us more relevant reasons about it.”
An animated video entitled: Hi. Did you miss me? I want to tell you that controlling your desires (lust) is what makes us humans apart from animals”. This is only for those who can understand hahaha (Posted on June 06, 2020)	“After this, feminists who do not understand will leave angry comments hihi”.	O you who believe (who are âmenû)! Let not one person mock (another) people. May be they (the mocked) are better than the others, and nor women (mock) at other women, may be they (the others) are better than themselves. And do not defame one another, nor call one another by nicknames. Evil is transgressed names after the Faith, and whoever does not repent, then such are indeed the wrong-doers. (QS. Al-Hujurat: 11)

## Discussion

The presence of @indonesiatanpafeminis.id (@indonesiawithoutfeminist.id) sheds light on an unpopular subject that may well be significant to observe through the lens of feminism discourse. The existence of digital space presents a platform for women from different groups with various interests throughout Indonesia. Some users provide important ideas on awareness toward various problems that women experience. On the other hand, the digital space could present various misconceptions about feminists and some content contend feminists’ views related to the local context.

This discussion explains how the digital space has the potential to provide room for discussions on various issues rarely discussed openly by women groups in Indonesia that supports for or against feminism groups. Based on the findings, it is necessary to look at the issues in the @indonesiatanpafeminis.id account and the debates in this digital space. Based on the various topics that arise it could be understood that all the issues and debates are affected by the local context.

In addition, this discussion also reflects on virtual spaces and the relationship between online and offline activities regarding the issues and socio-politic contexts about the appearance of the @indonesiatanpafeminis.id account. The strength of political fight in the parliament related to the ratification of the Sexual Violence Eradication Bill is assumed as the reason for the creation of the @indonesiatanpafeminis.id account. This fact provides a lesson for the feminist movement that digital spaces could be a political tool to make women as subjects objectified by political interests.

### The Potential of Social Media for Feminist Discourse

The research found that Instagram, as a social media, is utilized as a virtual space for the development of feminist issues, by both the pro-feminist and anti-feminist groups. Social media could facilitate various groups including women groups which can potentially provide a wider space. This phenomenon is one of the things that gave birth to the anti-feminist groups among pro-feminist groups. Moreover, the researched phenomenon has also revealed an interaction between these two opposing groups.

While many anti-feminist groups present themselves openly, there are also anti-feminist groups that decided to stay anonymous. The identities of the @indonesiatanpafeminis.id account’s admins are anonymous not revealing their data on the account. In their post, the admins have only appeared as animated images. There are also Muslim women groups that are relatively pro-feminist or raise feminist issues such as Aisyiyah Pusat, Muslimat NU, Rahima, and Perempuan KAMMI. These accounts are quite dominant and more popular among society than the @indonesiatanpafeminis.id account that is relatively new and anonymous. All the accounts have been around longer and have more followers than the @indonesiatanpafeminis.id.

According to an informant from National Commission on Violence Against Woman or Komnas Perempuan, @indonesiatanpafeminis.id account’s position as being anonymous confirms their focus on the issues, @indonesiatanpafeminis.id generally pay attention to the audiences’ comments. This account is different from, for example, Komnas Perempuan’s account who uses their media as a showcase to convey what they have done as an organization. It is the same with other accounts that use their organization’s account to convey more about their activities. However, the accounts that focus on issues such as the @indonesiatanpafeminis.id account target more general audiences to understand the issues based on their framework. In other words, in media that focus more on issues, the personal identity of the admins of their group or organization is not important (Interviewed on 25/5/21).

Referring to the issues raised by the @indonesiatanpafeminis.id account it is not a dominant discourse among Muslim women or Indonesian society. In general, Muslim groups such as Aisyiyah and Muslimat NU are part of the two biggest Islamic organizations in Indonesia, Nahdlatul Ulama, and Muhammadiyah. As a result, if we compare the comment in the @indonesiatanpafeminis.id account, generally more comments criticize their posts than comments that support them.

The ratification of the Law on the Elimination of Domestic Violence shows that the discourse related to the reality of domestic violence that befell women and kids has become an issue that is recognized by society and the government. The legitimation of this law is proof of the hard work done by the women’s movement or Indonesian feminists related to the women’s violence in domestic areas that still exist. Referring to the existence of the Law on the Elimination of Domestic Violence, women’s movement and law are important to ensure the prevention of domestic violence in Indonesia.

Regardless of the identity of women groups that are being shown in social media, these findings support the idea that women could be involved in raising their views and attitudes toward important issues. Social media allows women to be a creator and take part in public spaces, non-anonymous or anonymous. This is also emphasized in the statement that women could use their social media to create and distribute their knowledge to the public (Davis, 2018). Culturally, social media could remove barriers for women to communicate in public spaces that previously pushed women to be in domestic areas. In other words, the @indonesiatanpafeminis.id account as a social media could be presenting women from various groups in public spaces. Through social media, women could be presented in public spaces, to talk about their interests anonymously, without needing to leave their houses. They could even be triggering and be involved in the debates.

An interesting aspect about the communication activities in the virtual space of @indonesiatanpafeminis.id is that the admins are never involved in the debates between the pro and contra in their posts. Multi-study investigations conducted by Hall (2018) show that the use of social media is not equivalent to social interaction. When interaction happened through social media, the participants tend to chat or post on their walls. In this case, the participants are chatting in the comment section and it is an expression of their feelings. As if the admins have the role to raise the issues in their posts and let their audience debate whether they agree or disagree with the issues. Although we cannot deny that occasionally within the offending debates between the pro and contra emerge, we should also note that in the @indonesiatanpafeminis.id account there are also informative comments. The response explains the mistakes that the admins made in their posts, regarding both their concept in understanding, data accuracy, or their logic. Netizens gave their explanation about the issues posted by @indonesiatanpafeminis.id’s admins. On a post that talked about feminism push women nowadays to have the same role as men that posted on April 22, 2020, some netizens commented on the meaning of feminism. They said that “Feminism is a choice. So, if you want to embrace the feminist side or masculine side in yourself, it is up to you as long as you are not feeling oppressed. If there is a woman who feels oppressed to stay in a domestic setting because her environment enforces this on her, it is wrong. But if there is a woman who likes to be in a domestic setting because of her own will, it is okay. Feminism is all about choices” (Posted in @indonesiatanpafeminis.id, April 22, 2020).

Some comments explained the meaning of feminism and they thought that the admins do not understand feminism. In the same post, the admins also questioned Kartini’s title as a feminist because she practiced polygamy. Kartini is an Indonesian activist who advocated for women’s rights in the colonial era. One netizen explained about Kartini, “the reason why she (Kartini) opposed it (the practice of polygamy) is that she had been through it and was also a victim of it. This topic is not easy but the point is polygamy practice will likely be more of a torment for women and children. In the Al-Qur’an, the context of polygamy itself is not recommended because the practice does not exist, but from not controlled to be controlled. Along with the times, I think it is okay to not practice polygamy.” Another comment by netizen quoted Krisnina, the writer of Pikiran Kartini (Kartini’s Thoughts), that said, “one of the reasons why Kartini finally accepted to be married to a man who has wives or be in polygamy, is because of her great respect and love for her father" (Posted in @indonesiatanpafeminis.id, April 22, 2020).

In other words, @indonesiatanpafeminis.id with their posts has opened a virtual space for everyone to get involved, either for individuals or groups that reject and do not understand feminism or for individuals or groups that do support and have a better understanding of feminism. A political observer stated that, quoted “there will be debates among public opinions that will make people rethink feminism, and unfortunately in Indonesia when controversial matters arise critical thinking is not deemed as common practice in our culture. When controversy arises the first thing we do is sharpen our identity and inevitably it comes to ‘you do you, I do me’ if you are not part of us then you are the enemy. As a result, the public opinion debates make people rethink feminism, and unfortunately in Indonesia, this critical thinking is not something that we always do when there is a controversial issue. When controversial issues arise, the first thing we do is to sharpen our identity and, in the end, if you are not a part of our group, then you are our enemies” (Interviewed on 31/5/21).

Regarding the debate that happened in the @indonesiatanpafeminis.id account, a commissioner of Komnas Perempuan stated that this is something that could not be avoided. As an account that focuses on the issues, @indonesiatanpafeminis.id focused on the issues and the comments from their audiences. They raised the simple issue of questioning feminism in general and even asked what is wrong with feminism. However, these issues are also attracting general audiences that have no previous knowledge about feminism and want to know about feminist issues from various views (Interviewed on 30/5/21).

One of @indonesiatanpafeminis.id’s followers said that she was attracted to see the account because she thinks “that’s good. We have optional choices. Why not? And sometimes what they said is true.” In other words, feminist content in the @indonesiatanpafeminis.id account raised a negative way of thinking about the feminist movement and also ignored various views about feminism. On the other hand, this is also a chance for feminists to explain and correct the wrong way of understanding feminism. However, how they communicate with others is just as important to determine whether the explanation from feminists might lead to a new understanding or only generate conflicts.

This opinion is in line with a follower’s opinion that does not agree with a post in the account. She continued to say that “they sarcastically deliver their messages. Sometimes it does not make sense.” Even though the informant does not agree with the statements or contents from @indonesiatanpafeminis.id account, she expressed her disagreement with the way their admins communicate in their account. She said, “some statements (comments) that I saw are not ethical. We should be able to share our opinion only, not personally attack anyone.”

This opinion is understandable because the way they communicate appears as though they are attacking others, giving the impression that feminists always feel superior. One of the followers of this account commented, based on the content and dialogue, the interviewee feels that “feminist exudes superiority, not equality.” This opinion needs to be acknowledged not only in Indonesia, for example, Baumeister (2007) thinks that early feminists tended to prefer gender equality but today’s feminists tend to favor the superiority of women over men, feminism is mostly hijacked by male haters. In correlation to what happened in the virtual spaces of @indonesiatanpafeminis.id, then when communications in virtual spaces portray feminists as less appreciative toward others’ opinions or degrade others’ opinions, it strengthens the bad impression of feminists. This may mitigate the potential of virtual spaces to become public spaces for women to express their thoughts and views.

However, it is proven that the @indonesiatanpafeminis.id account could raise the feminist discourse in social media. One of the followers in an interview said that she was attracted to the account after she saw one repost from a story and at that time, the content is popular among women’s rights activists. This means that the @indonesiatanpafeminis.id account succeeds in attracting people, both who are pro and those in contra with their contents. The existence of feminist discourse in public is needed and is one of the alternative ways from the marginalization of feminism issues in the media.

### The Contextualization of the Offline–Online Feminist Movement

Based on the analysis of online and offline relations, it is found that the influence of socio-politic situation is related to the appearance of the @indonesiatanpafeminis.id account, especially about public issues related to the Sexual Violence Eradication Bill.

The research found that the admins of @indonesiatanpafeminis.id account do not provide information as an organization, association, or having links with other organizations. However, the informant from Komnas Perempuan (National Commission on Violence Against Woman) said the unclear identity of the admins of this account shows a stronger work mechanism. Accounts such as @indonesiatanpafeminis.id are connected to a bigger ecosystem with a specific political agenda and ideology. The attacks on feminists related to the Sexual Violence Eradication Bill are connected to the first academic script of the same bill that contains feminist legal theory as the base. In the end, with some considerations, the bill uses the critical legal theory as the base. She added negotiations were conducted for the bill to be accepted and officialized to save more women from becoming victims (Interviewed on 30/5/21).

As a result, the same informant stated that the Sexual Violence Eradication Bill was already adjusted to various demands even though it still keeps substantial things such as rejecting adultery clauses to be included on the bill. The commissioner stated the reason for the rejection is because substantially, the adultery issues could not be categorized as sexual violence. Even though Indonesian society thinks of it as an issue, the truth is it is a different issue. The different opinions are relevant to the emergence of accusations about feminists as free-sex followers as shown on the @indonesiatanpafeminis.id account. One of the posts shows an animation of a woman who analogized feminists as animals because they could not control their (sexual) desire as free-sex follower stigma (even though it is not expressed directly) (Posted in @indonesiatanpafeminis.id, October 28, 2020).

Opinions that view what @indonesiatanpafeminis.id has done as something premeditated and organized are in line with the political observer’s opinion. He stated, “was it planned, yes because they are programmatic, they learned from previous experiences. Even if this was not conducted by the same people, at least it was conducted by people within the same political spectrum, so they learned and have a ‘dog whistle,’ where jargons they spread will quickly be identified by those that have the same understanding or ideology causing it to become massive spreading through the social media” (Interviewed on May 31, 2021). This is in line with the emergence of similar accounts but using the identity of big cities in Indonesia, such as @jakartatanpafeminis2, @padangtanpafeminis_ and @bandungtanpafeminis. These accounts repost content from @indonesiatanpafeminis.id or create new content with the same ideology.

Referring to the existence of various Muslim women accounts, the existence of the @indonesiatanpafeminis.id account could be said that the account itself still contains and uses the understanding of women’s position in patriarchy values. Their interpretation of feminist is related to women in Islam; in most cases, they raise issues about gender-biased matters and ignore the gender-friendly interpretation that forms the basis for Islamic women movements such as Aisyiyah, Muslimat NU, Perempuan KAMMI, and Rahima. Women’s position is being constructed with the idea that women are more valuable in the domestic area. Women’s existence in public areas is often interpreted as the cause or *raison d’etre* for sexual violence. This idea is in line with the assumption that when men raped women, the individuals tend to justify and rationalize their violence by maintaining the myth and ideology of blaming the victim and believe that the victim did something to attract the violence (Anderson, 2015). In an interview, the informant from Komnas Perempuan stated there is a growing number of groups nowadays that are supporting or accepting of women in the public spaces as long as they are obedient and position themselves as a subordinate under men (Interviewed on 30/5/21). Based on that observation, the emergence of the @indonesiatanpafeminis.id account is being understood as an effort to give women space to be present in public space but they also need to respect the patriarchal values. In the reformation era, opportunities for women to exist in public spaces appear more than before, not only as a government’s “tool” but also as an individual agent for development. This is also supported by ‘‘Instruksi Presiden” (President’s Instruction) no. 9 September 2000 about Mainstreaming Gender in National Development (Hasan, Anugrah, and Pratiwi, 2019).

The relation between offline and online activities could be understood from the emergence of the family issues in a few posts from @indonesiatanpafeminis.id. The existence of women in domestic spaces is being related to the issues about a family whose prosperity is being charged to women. In Indonesia, there is also a women’s group called Aliansi Cinta Keluarga (the Family Love Alliance) in Indonesia (AILA) who is very against feminist because they think that feminist hates family. The @indonesiatanpafeminis.id account also shared the same statement. On June 13, 2020, they posted a short video that talks about how feminist hates the family concept because they could not get enough love from their own family. The existence of this group is related to the foundation of Sekolah Perempuan (School for Women) established in several cities in Indonesia. Based on the information from one informant, one of the reasons behind the foundation of Sekolah Perempuan is the increasing number of divorce cases. It means that women are considered to need special education to avoid divorce in their families.

The resistance toward feminism, based on various contents that have been collected and analyzed from the @indonesiatanpafeminis.id account, is caused by several reasons such as the fear for things that come from the West, the lack of knowledge about feminism and feminism in the women’s movement, patriarchy values, and matters related to the gender-biased interpretation in Islam. The fear for things that come from the West, overlook the idea of equality or human rights could not be said as West values. This claim ignores the fact that many women are not white and fighting for their rights, both in western countries or in other places (Bulbeck, 2002). In Indonesia, one of the most frequently mentioned fears is the assumption that feminist ideas, thoughts, and movements do not have social and cultural roots in Indonesia’s society (Arifia and Subono, 2017).

Contents about feminism in the @indonesiatanpafeminis.id account present various negative perspectives. However, these perspectives are not new. Baumeister (2007) think that early feminist tends to like gender equality more but feminist nowadays tends to like the superiority of women over men, feminism is hijacked more by men haters. The feminist’s beliefs often attack society’s thoughts and practices that are considered normal or right. Therefore, the feminist is often seen as an identity that is being stigmatized by things that are considered contrary to the existing values or things that are bad (Dye, 2005). The diversity of feminist thoughts that evolved globally is being interpreted equally by @indonesiatanpafeminis.id. Although as stated by one of the Indonesian feminist experts “feminism is *glocal*, global but at the same time local. The meaning of global here is because patriarchal culture is global, this culture is strong everywhere. However, it is also local. Like in Indonesia, the problems that women face in Indonesia are different from the problems in America or Europe. In Indonesia, we face other Muslims as problems by how texts have been interpreted through every patriarchy lens. Therefore, patriarchal values exist. Feminism fights to counter or critique the interpretation of it” (Interviewed on 18/5/21). Indonesians are generally bound by religious rules in every aspect of their thought and activities throughout their life. In regards to religious interpretations, a political observer explained in an interview, “The diversity of feminist movements in Indonesia may well have been created by different interpretations (*tafsirs*) of feminist views and views on the *Syariah* or texts in the Al-Quran. The war of interpretations is interesting because inevitably it will create new discourse or even give birth to new thoughts on feminists in Indonesia” (Interviewed on 31/5/21).

Bad stigma is related to feminism that contradicts cultural traits related to the family such as how the ideal role as a wife is supposed to be, children’s education in the family, and also abortion issues that are related to religion. This negative stigma is also portrayed in posts by @indonesiatanpafeminis.id which revealed their hatred or attempts to harass feminists. The attempt to harass each other happened to the point of using harsh words or statements full of hatred (to provoke feminists). That mockery was then responded by one of the pro-feminists with a quotation of a verse from the Al-Qur’an about the disrepute of someone who mocks others (QS Al-Hujurat:11). On June 6, 2020, a video post (in @indonesiatanpafeminis.id, June 06, 2020) by one of the admins stated the difference between humans and animals is how to manage their lust. She mocked feminists because she thinks that feminists could not control their lust. This comment is one method to fight the stigma about pro-feminists who are anti-family, anti-religion, and express deviant behaviors. The interpretation of feminist that specializes experience as a tool for “imagining justice” of text that produces gender inequality, Amina Wadud quoted the difference between the idea of Al-Qur’an and contemporary as social justice to show that “Muslim women create their voices when they experienced text” and so she is referred to as an “experience authorities” (Seedat, 2013).

The netizen’s effort to fight the contradiction between feminist and religion also involves arguments taken from *surahs* in the Al-Qur’an and the histories of Islam. It means this debate could be interpreted as when the women’s movement clashes with Islam, most of the conflicts that happened related to the meanings built by some believers with their ideologies. This is different from what was done by professional women that emphasize issues related to the reality happening within the society such as poverty that marginalizes women, women reproduction health issues, domestic violence, and supporting women to excel in the educational area, and also being involved in public areas. However, these efforts are not without critics because the political economy study also has suspicions toward efforts of women development in public areas, both through education or employment, but in the end, it makes women turn into the consumer in consumerism culture (Genz, 2006; McRobbie, 2009).

The Muslim women in Indonesia are apparently more open to accepting different views in the practice of their religion such as whether or not female Muslims to wear hijab. This perspective is also portrayed in the @indonesiatanpafeminis.id account when they posted one of their admins who do not use the hijab (Posted in @indonesiatanpafeminis.id, July 11, 2020). This woman is portrayed in an animation version as a young woman with long blonde hair wearing glasses and a hat. It means that several things could be agreed upon by Indonesian women groups and several things could not be agreed upon. As a result, this space also portrays the Indonesian women’s movement that fights for women’s rights yet also resists certain matters; therefore, negotiations are needed to resolve these issues.

The research found that the actors behind the @indonesiatanpafeminis.id account emerge as individuals but carry the same political views as men’s interest, with both religious argument and normative value (patriarchy). Women’s groups exist as a subject that managed to break through from resistance issues but on the other hand unconsciously positioned themselves as an object. They become an object when their existence is only to fulfill men’s desire for marriage. In other words, their existence is unconsciously being used as a tool to fight for men’s interests, such as in polygamy issues, sexual violence, and related to the enmity of political parties in parliament. This is one of the issues that Simone De Beauvoir (2014) said about the relationship between women and men. The most important thing that women need to fight for is not their rights but the understanding of their clarity as women (De Beauvoir, 2014). On the contrary, when women have freedom, they will still be positioned as an object and place men as the subject. This happens with women’s groups behind the @indonesiatanpafeminis.id account. Through their understanding as a subject, women must have an intersubjective relation with men that see themselves as subjects and see others as a subject too (De Beauvoir, 2014). This view is a key to condemn the accusation from the @indonesiatanpafeminis.id account about feminist as being a woman who hates men and carries a mission for equality.

Based on actual occurrence in society, equal opportunity between men and women has already been implemented in a few educational institutions. The number of students in undergraduate programs at Indonesian universities is relatively balanced between female students (3,154,179) and male students (3,417,142) (Ministry of Research, Technology and Higher Education, 2018). However, some women face the dilemma in regards to their roles as wives and mothers after they graduated. Culturally, women are required to be the ones who bear the great responsibility to do house chores and take care of their children. In Indonesia, many men are being well educated by their parents but are not given the responsibility to do any house chores. Some women in Indonesia who come from upper-middle-class families are pampered by having lower-middle-class women as housemaids or assistants. Others are helped by having female relatives, such as their mother, sisters, or other relatives, who take on the burden of some household chores. This condition is one of the reasons why @indonesiatanpafeminis.id still posts the issues about equality and family.

## Conclusion

To conclude, the researchers gather that the existence of social media plays an important role in creating a virtual discussion about feminism and anti-feminism, even for the users that are not familiar with these issues. Feminists, as well as anti-feminist, have access to use the platform and make content that represents their awareness as an individual, social movement as well as a political movement. As an account that focuses on many issues, @indonesiatanpafeminis.id (@indonesiawithoutfeminist.id) also provides space to have an open debate or discourse on several important issues among netizens from those who deeply understand feminism to those who outright reject feminists. It is proven that the @indonesiatanpafeminis.id account could raise the feminist discourse in social media.

In Indonesia ideas on anti-feminism of @indonesiatanpafeminis.id are less popular compared to feminist movements promoted by other Muslim women’s movements. Those Muslim women’s movements are Aisyiyah, an Islamic organization formed by Muhammadiyah, and Muslimat NU, another large organization by Nahdlatul Ulama, and also Rahima (Center for Education and Information on Islam and Women’s Rights). These organizations are considered progressive women’s organizations that have been collaborating with the National Commission on Violence Against Woman (Komnas Perempuan). Anti-feminist issues raised in the virtual space via Instagram by women’s groups with Muslim identities (@indonesiatanpafeminis.id) in the end cannot ignore the movement of Muslim women in the offline space that has been developed and people’s assumption toward their connection to certain political interests.

The existence of @indonesiatanpafeminis.id has the potential to strengthen anti-feminist issues related to political or patriarchal interests in society. This tendency can occur with the use of the identity of big cities in Indonesia in accounts that affirm their rejection of feminists, namely, @jakartatanpafeminis2, @padangtanpafeminis_ and @bandungtanpafeminis.

Thus, there is a need for further research to explore the links between anti-feminist groups and Islamic political group movements that may have emerged since the reform (1998). There is also a need to understand how far women politicians are aware of issues on women’s interest and their struggles in the political arena. In addition, further studies are needed to explore how feminist groups and anti-feminist groups organize themselves in digital media. Furthermore, we have to elaborate more about the virtual debates between both sides as a digital feminist discourse that could be used to criticize and develop women’s movement through online and offline dynamics and to interpret feminists in a country with multicultural contexts.

## Data Availability

The raw data supporting the conclusion of this article will be made available by the authors, without undue reservation.
